# Structural Studies of the Taurine Transporter: A Potential Biological Target from the GABA Transporter Subfamily in Cancer Therapy

**DOI:** 10.3390/ijms25137339

**Published:** 2024-07-04

**Authors:** Dorota Stary, Marek Bajda

**Affiliations:** 1 Department of Physicochemical Drug Analysis, Faculty of Pharmacy, Jagiellonian University Medical College, Medyczna 9, 30-688 Cracow, Poland; dorota.stary@doctoral.uj.edu.pl; 2 Doctoral School of Medical and Health Sciences, Jagiellonian University Medical College, św. Łazarza 16, 31-530 Cracow, Poland

**Keywords:** SLC6A6, taurine transporter, structure, docking, homology modelling, molecular dynamics, cancer

## Abstract

The taurine transporter (TauT, SLC6A6) is a member of the solute carrier 6 (SLC6) family, which plays multiple physiological roles. The SLC6 family is divided into four subfamilies: GABA (γ-aminobutyric acid), monoamine, glycine and neutral amino acid transporters. Proteins from the GABA group, including the taurine transporter, are primarily considered therapeutic targets for treating central nervous system disorders. However, recent studies have suggested that inhibitors of SLC6A6 could also serve as anticancer agents. Overexpression of TauT has been associated with the progression of colon and gastric cancer. The pool of known ligands of this transporter is limited and the exact spatial structure of taurine transporter remains unsolved. Understanding its structure could aid in the development of novel inhibitors. Therefore, we utilized homology modelling techniques to create models of TauT. Docking studies and molecular dynamics simulations were conducted to describe protein–ligand interactions. We compared the obtained information for TauT with literature data on other members of the GABA transporter group. Our in silico analysis allowed us to characterize the transporter structure and point out amino acids crucial for ligand binding: Glu406, Gly62 and Tyr138. The significance of selected residues was confirmed through structural studies of mutants. These results will aid in the development of novel taurine transporter inhibitors, which can be explored as anticancer agents.

## 1. Introduction

The taurine transporter (TauT, SLC6A6) is a member of the solute carrier 6 (SLC6) family, which comprises proteins that transport molecules and ions across the biological membranes. The SLC6 family is divided into four subfamilies: GABA (γ-aminobutyric acid), monoamine, glycine and neutral amino acid transporters. TauT (SLC6A6) along with GAT1 (SLC6A1), GAT2 (SLC6A13), GAT3 (SLC6A11), BGT1 (SLC6A12), and CT1 (SLC6A8) constitute the GABA transporter subfamily [1]. Notably, the creatine transporter pseudogene (CT2, SLC6A10) is also classified within this subfamily, although its physiological role remains unclear [2] (Table 1).

Proteins from the γ-aminobutyric acid (GABA) subfamily are primarily considered therapeutic targets for central nervous system disorders such as epilepsy, depression, anxiety and pain. Currently, only one compound, tiagabine, a GAT1 inhibitor, is used in the therapy as an anticonvulsant drug [4]. The association between the main substrate of SLC6A6, taurine, and neurological diseases, as well as taurine’s protective role in the retina, have been previously described [4,5,6]. It has been suggested that decreased TauT activity caused by mutations, such as Gly399Val and Ala78Glu, is involved in retinal degeneration and dilated cardiomyopathy [6,7,8,9]. In addition to neurological disorders, selected transporters could serve as potential biological targets for anticancer agents.

**Figure 1:**
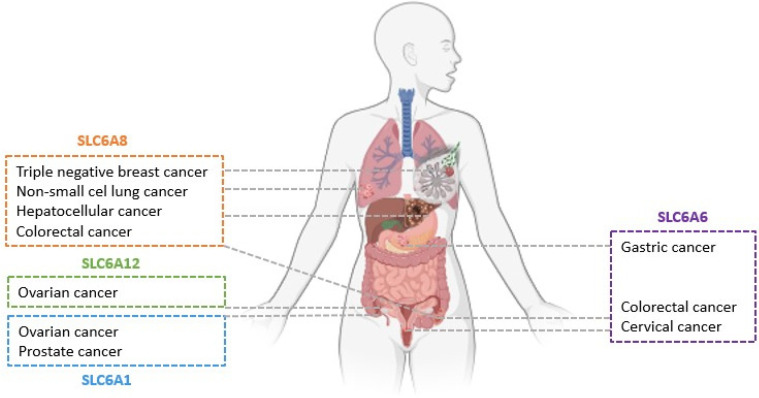
Overexpression of GABA transporters is associated with several types of cancer.

Increased activity and overexpression of TauT, as well as other proteins from the GABA group, have been detected in several types of cancer [10,11,12,13,14,15,16,17,18,19,20] (Figure 1). A high level of taurine transporter has been documented in colorectal (CRC), gastric (GC), and cervical cancers (CC) [13,14,15]. Overexpression of the SLC6A6 has been associated with CRC progression, increased survival and antiapoptotic effect on the cancer cells [14]. TauT has been indicated as a novel CRC-specific surface marker [14]. Furthermore, elevated TauT expression in gastric cancer was associated with a poor prognosis, and advanced, invasive tumor stage. Similarly to CRC, SLC6A6 has been proposed as a potential diagnostic marker for GC [13]. Additionally, TauT has shown elevated expression in cervical cancer tissue, although its precise role in cancer development requires further investigation [15]. Other transporters from the GABA subfamily could also serve as biological targets for anticancer agents: overexpression of SLC6A8 has been associated with CRC, triple-negative breast cancer, hepatocellular and non-small-cell lung cancers, as well as lung adenocarcinoma [16,17,18,19,20]. SLC6A12 has been implicated in the increased invasion of ovarian carcinoma cells [12], while SLC6A1 overexpression has been noticed in ovarian and prostate cancers [10]. Detailed function and role of taurine transporter in cancer development were discussed previously [21]. These data suggest that protein carriers such as taurine transporter could be a promising biological target in oncological therapy, particularly for colon cancer, one of the most common neoplasms worldwide [21]. It is worth mentioning that one inhibitor of CT1 is currently under investigation in the first stage of clinical trials against CRC [22]. Similarly, we propose the development of novel taurine transporter inhibitors and the evaluation of their anticancer activity, especially against colorectal cancer cells. Considering the limited availability of SLC6A6 transporter inhibitors, we believe that novel ligands could be identified based on the structure of the biological target.

Transporters from the SLC6 family, including SLC6A6 share the same overall spatial structure, and mechanism of action. Moreover, all GABA subfamily members require sodium and chloride ions for their activity [23,24,25,26,27,28]. Transport stoichiometry for TauT as well as GAT1, GAT2, GAT3, and CT1 was determined as 2Na^+^:1Cl^−^:1 substrate molecule [29]. A reduction in sodium levels leads to impaired taurine transporter function [30].

**Figure 2:**
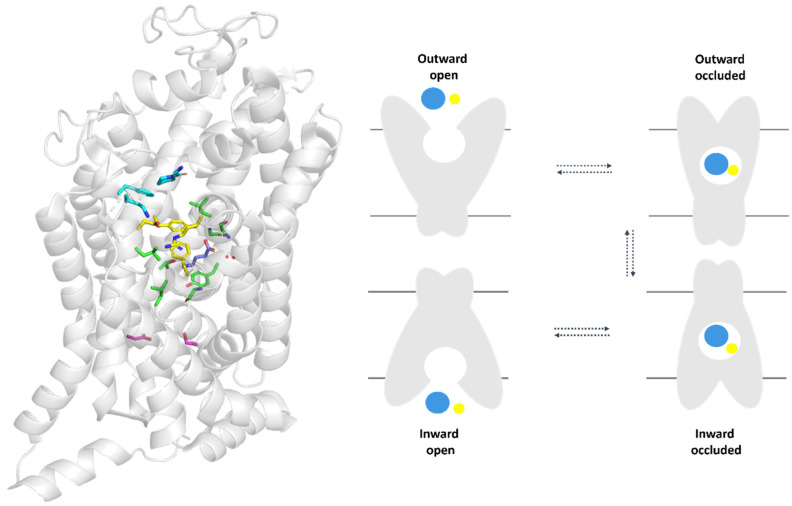
Topology diagram of the transporter from SLC6 family—spatial structure of SLC6A1 (GAT1) transporter with selected residues (**left panel**). Residues color: blue—S2 site, yellow—extracellular gate, green—S1 site, and pink—intracellular gate. Schematic representation mechanism of action of the transporter (**right panel**). Blue sphere—substrate; yellow sphere—ions.

Proteins from SLC6 family undergo four main conformational states during substrate transport: outward-open, outward- and inward-occluded, and inward-open (Figure 2, right panel). Under normal physiological conditions, these proteins uptake substrates and ions from the extracellular environment and release them into the cytoplasm. However, in certain physiological or pathophysiological conditions, such as those mimicking ischemia, the direction of the transport can be reversed (Figure 2, right panel) [31].

The overall structure of TauT is consistent with other proteins from GABA transporter subfamily (Figure 2, right panel); however, the exact structure remains unknown. Understanding its structure could facilitate the development of new ligands. Therefore, we decided to fully describe the selected protein. Analysis and characterization of the SLC6A6 transporter were achievable using data from Uniprot [32] and Protein Data Bank (PDB) [33]. The obtained structural data on taurine transporter were compared with the experimental structure of GAT1 [34], as well as models of GAT1, GAT2, GAT3 and BGT1 described in the past [23], along with information about TauT and CT1 found in the literature.

Here, we present for the first time the general structure of SLC6A6 obtained through homology modelling and the binding mode of TauT substrates and inhibitors. Our studies provide insights into the structural features of TauT and identify key residues involved in ligand binding.

## 2. Results and Discussion

Computational methods are widely used in the drug design and development. Homology modelling is one of the in silico methods that help determine the three-dimensional (3D) structure of proteins. Using models of biological targets, we can predict ligand-protein interactions and enhance new drug development [35]. In our study, we employed homology modelling to generate 3D structural models of the taurine transporter. We conducted sequence alignment, model building and scoring to obtain the best models of taurine transporter. These models were then used for docking studies of substrates and inhibitors. Additionally, we performed molecular dynamics simulations for the selected complexes to check their stability.

### 2.1. Model Building and Evaluation

Transporters from the SLC6 family present similarities in the amino acid sequences and share the same general 3D structure. Initially, we compared the amino acid sequences of proteins from the GABA subfamily with those of potential templates deposited in the Protein Data Bank (PDB) [33]. The TauT transporter revealed the highest identity and similarity with the *h*GAT1 transporter, with values of 47% and 63%, respectively (Table 2). The TauT protein shared approximately 58% similarity with *d*DAT (*Drosophila melanogaster* dopamine transporter), consistent with other GABA transporters member (Table 2).

We noticed that bacterial homologue LeuT (Leucine transporter) had a less consistent amino acid sequence with SLC6A6, nevertheless, it is sufficient for homology modelling studies. In addition to similarities in sequences, similarities of ligands of the transporters are important. The substrate of TauT, taurine, had an amino acid core, making it structurally closer to leucine than to dopamine or serotonin, which are monoamines. Based on these findings, we decided to use as templates all proteins from SLC6 family deposited in the PDB database. Our study aimed to generate models of TauT in different conformational states, which is necessary for proper discussion of substrate transport and inhibitor binding. For homology modelling, we selected 14 experimental structures which represent outward-open, outward-occluded, inward-occluded and inward-open conformation. As templates we selected: structures of *Aquifex aeolicus* LeuT, *Drosophila melanogaster* DAT, human SERT, GlyT, and GAT1 (Table S1). Obtained models were evaluated with several scoring functions. We found that the best models for taurine transporter in outward-open and occluded states were built on the DAT templates, PDB code: 4XP9, 4XP4, 4XPH and 6M2R. Best models in inward-open and occluded conformation were built based on the structures of GlyT1—6ZBV; and GAT1—7Y7Z and 7Y7W. Among LeuTs, the most suitable templates for model construction were: 4MMB and 3F3A (outward-open state), 2A65 and 2Q6H (outward-occluded state). Selected models and their corresponding score values are presented in Table S2.

### 2.2. Structure of Taurine Transporter

#### 2.2.1. Overall Structure

**Figure 3:**
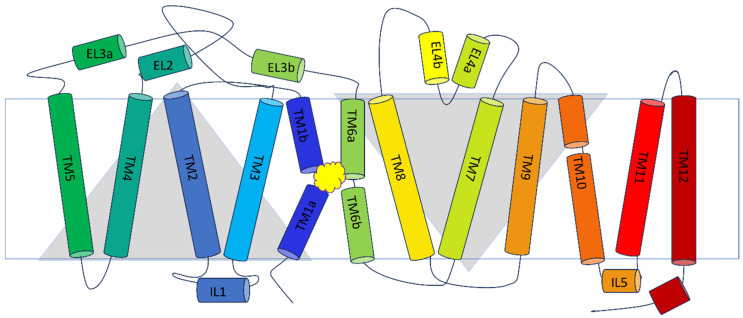
Spatial structure of SLC6A6 transporter. Yellow cloud marks the ligand binding site.

Proteins counted to the SLC6 family, including the taurine transporter and other GABA transporters, consist of 12 transmembrane helical domains (TMs), with N- and C-termini located intracellularly. The core of the transporters is formed by domains TM1 to TM10. Helices TM1 to TM5 and TM6 to TM10 are linked by a pseudo-two-fold axis. The extracellular surface of the taurine transporter is formed by three long loops: EL2, EL3 and EL4. Notably, loop EL4 contains helical fragments, creating a V-shaped structure. It was observed that TauT possesses a longer EL2 loop than the bacterial homologue LeuT, but similar to the GAT1 and GlyT1 [36]. The IL1 and IL5 loops interact intracellularly (Figure 3) [34]. The key domains for TauT ligand binding are TM1 and TM6, which possess the non-helical fragments in the middle of the cell membrane. The same observations were made in the case of glycine and leucine transporters [34,37]. The TM1 and TM6 together with TM3 and TM8, form the S1 site, where ligands bind. As TauT activity is conjugated with ion concentration gradient, the ion binding sites can be found. They are located between TM1, TM2, and TM6–TM8, similar to other transporters from GABA subfamily [21,23,26,29,38,39].

**Figure 4:**
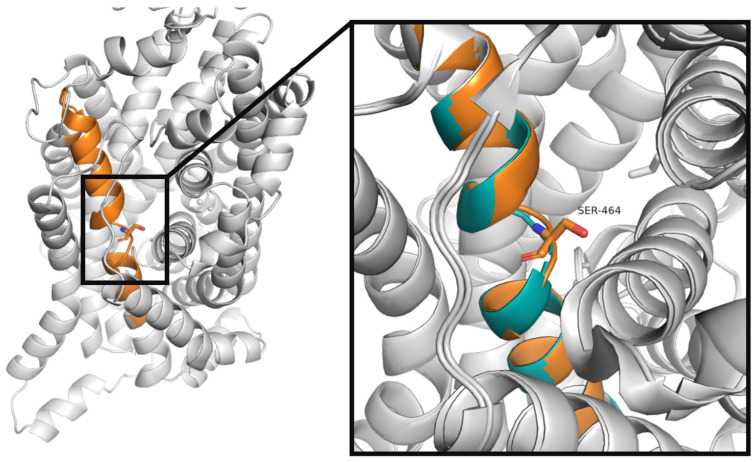
The overall structure of the taurine transporter in an inward-occluded state (**left panel**). Superposition of SLC6A6 model and GAT1 structure (PDB code: 7Y7W). View on the Ser464 in the TM10 domain from SLC6A6 (orange stick and cartoon), TM10 from GAT1 (green cartoon) (**right panel**).

In TauT, like in all GATs, an additional residue—serine (Ser464 according to SLC6A6 numbering)—is found in TM10 near the S1 binding site. The presence of this insertion poses a challenge for modelling. Furthermore, transporters from the GABA subfamily have different neighboring residues in these sequence fragments: Ala-Ala-Ser-Gly (TauT, GAT2, GAT3), Ser-Ala-Ser-Gly (GAT1 and CT1) and Ala-Ser-Ser-Gly (BGT1) [23]. Colas et al. discussed the function of Ser479 in CT1, suggesting that it formed an unwound region or π-helix [25]. To determine the arrangement of Ser464 in TauT, we generated models of the transporter and compared them with selected structures of GAT1 in both inward-occluded and inward-open conformations (PDB code: 7Y7W and 7Y7Z, respectively) as well as with other SLC6 family members. Our analysis suggests that this insertion forms a non-helical fragment in a taurine transporter, similar to the one in experimental structures of GAT1 (Figure 4).

#### 2.2.2. Mechanism of Action

**Figure 5:**
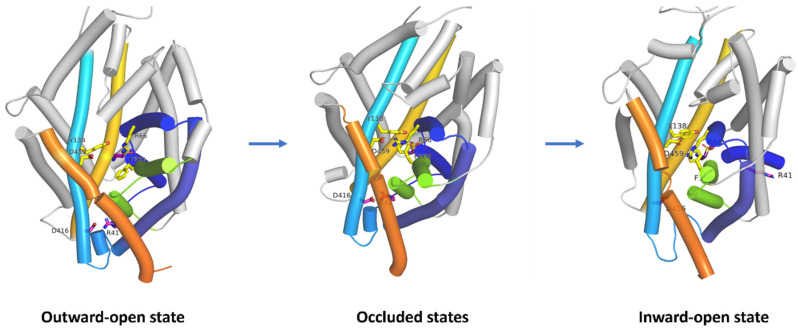
Schematic representation of taurine transporter conformational changes during transport: outward-open conformation (**left panel**), occluded states (**middle panel**), inward-open conformation (**right panel**). Occluded states represent average structure for outward-open and inward-open conformation, due to small-distance changes in domains and residues between each other. Domains shown as cartoons, colored as follows: TM1—dark blue, TM2—marine, TM3—light blue, TM6—green, TM8—yellow, and TM10—orange. Yellow sticks—residues from the extracellular gate, pink sticks—residues from the intracellular gate, and purple—taurine. For clarity, TM11, TM12, ions and ligands are not shown.

Proteins from the SLC6 family, including SLC6A6, undergo four main conformational states during substrate transport: outward-open, outward- and inward-occluded, and inward-open (Figure 2, right panel). We performed a detailed analysis of SLC6A6 to describe changes of domains and residues during the transport process (Figure 5).

The observed effects are common for the whole SLC6 family [39]. During transport domains TM6, TM1, and TM2 shift toward TM3 and TM10 domains. The most extensive shift appears in the arrangement of the TM1 domain. In the inward-open state, this domain is bent by nearly 45°, compared to its position in the outward-open state (Figure 5, left and right panel). The most noticeable are movements of residues from extracellular and intracellular gates. In the outward-open conformation, the transporter’s extracellular gate is open. The distances between aromatic rings from Tyr138 (TM3) and Phe300 (TM6) is approximately 12 Å, while the distance between a guanidine group of Arg66 (TM1) and an acidic moiety of Asp459 (TM10) is approximately 7 Å. These distances allow the substrate and ions from the extracellular environment to enter and bind to the transporter’s active site. In this state, the intracellular gate is closed, and Arg41 and Asp416 residues interact via a salt bridge at a distance of 3.5 Å (Figure 5, left panel). As ligands interact with amino acids from the binding site, residues from the extracellular gate move closer to each other. Arg66 forms an ionic bond with Asp459 while Tyr138 interacts with Phe300 through hydrophobic interactions. Additionally, the side chain of Phe300 rotates approximately 90°. When the transporters occur in the occluded states (outward-occluded and inward-occluded), substrates and ions are blocked inside the protein (Figure 5, middle panel). In these two conformations, we observed small shifts of TM1, TM2 and TM6 domains, which make outward-occluded and inward-occluded states closer to outward-open or inward-open conformations, respectively. The distances between residues from extracellular gate are similar: Tyr138 and Phe300—near 6 Å; and Arg66 and Asp459—near 3 Å. Difference occur in the case of intracellular gate. In outward-occluded state, Arg41 and Asp416 are in the same positions that in the outward-open state. When transporter adopts inward-occluded conformation, interaction between Arg41 and Asp416 breaks and the distance between residues is growing. In the last step of transport, SLC6A6 adopts the inward-open state with open intracellular gate. Substrate and ions can then be released into the cytoplasm of the cell. Meanwhile, the extracellular gate is closed with the 7 Å distance between Tyr138 and Phe300 rings, Arg66 forms a salt bridge interaction with Asp459 at a distance of 3 Å. The entrance to the S1 binding site of the transporter from the extracellular side is blocked (Figure 5, right panel).

#### 2.2.3. Binding Sites

The overall structure of the taurine transporter is similar to those of other proteins from the SLC6 family (Figure 3). Sequence alignment for SLC6 family members allowed us to point out differences between proteins, with special interest in residues that belong to the extracellular gate and S1 site (Table 3). Residues creating ion binding sites and intracellular gate are mostly conserved among family members. The S2 site, located in the extracellular vestibule of the transporter, represents the space where ligands can also bind, either wholly or partly [40]. In the case of TauT, the S2 site is created by: Gly366, Pro367, Phe455, Gln456 as well as four residues counted to the extracellular gate. Almost the same residues in the S2 site were observed in other transporters from GABA subfamily. Only Gln456 from TauT is substituted by Lys448 in GAT1.

The extracellular gate of TauT is formed by two pairs of residues: Tyr138–Phe300 and Arg66–Asp459. The same pairs of residues are also observed in GATs (GAT1, BGT1, GAT2-3) and CT1. For instance, in GAT1, the effects of mutations in the extracellular gate on the substrate transport have been studied. It was observed that mutations of arginine and aspartic acid to lysine and glutamic acid, respectively, resulted in the loss of GABA transport [23]. Although a similar study has not been performed for TauT or CT1, it is obvious that the extracellular gate is crucial for the activity of all transporters. Alignment of the GABA transporter subfamily sequences revealed differences in the S1 site, where ligands bind. In the taurine transporter, the S1 site is created by: Gly57, Phe58, Gly60, Leu61, Gly62, Leu134, Ala303, Leu306 and Glu406. Residues from extracellular gate: Phe300 and Tyr138 are also counted to the S1 site, although due to its gating function they were described earlier. Small, neutral residue Gly57, from TauT, is replaced in other GABA transporters by aromatic residues (Tyr60 in GAT1, Phe58 in the CT1) or glutamic acid (Glu52 in BGT1, Glu48 in GAT2 and Glu66 in GAT3). The importance of this residue was confirmed by mutagenesis studies: Gly57Glu lost transporting function, showing a 99.8% reduction in [^3^H]taurine uptake [28]. Substitution of Leu306 in TauT by Gln (originally from BGT1) decreased taurine affinity and increased GABA [28]. Glu406 seems to be the most important. In other transporters, it corresponds to Thr400 (GAT1), Cys399/394/414 (BGT1/GAT2/3), and Gly421 (CT1). Mutagenesis studies showed that Glu406Cys substitution leads to an increased affinity for GABA. Possibly, Glu406 plays a significant role in taurine recognition [28]. Docking studies on the SLC6A6 mutant confirmed that this acidic residue is essential for substrate recognition.

GABA subfamily transporters are sodium and chloride dependent [29]. Residues which bind ions are mostly conserved in the whole subfamily, and in TauT the ion binding pockets are created by: Phe58, Asn63, Ser301, Asn333 (Na1 binding site), Gly56, Val59, Leu398, Asp401, Ser402 (Na2 binding site), Tyr83, Gln297, Ser301, Ser337 (Cl binding site). Two differences between TauT ion binding sites and other GABA subfamily transporters were observed. The large, aromatic amino acid Phe58 of TauT corresponds to small alanine residues, Ala61 and Ala69 in GAT1 and CT1, respectively, while in BGT1 and GAT2-3, it is substituted by isoleucine. Experimental studies showed that the Phe58Ile mutant exhibited a reduction in [^3^H] taurine uptake by 58.1% [28]. Further, Val59 in TauT is changed to isoleucine residues in GAT1–3 and BGT1.

#### 2.2.4. S1 Binding Site Analysis

**Figure 6:**
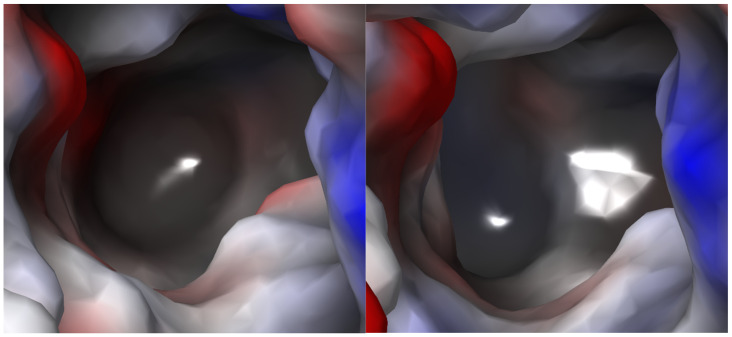
Electrostatic potential maps for the S1 binding site of taurine (**left panel**) and creatine (**right panel**) transporters. Red color—accumulation of negative electrostatic potential; blue color—accumulation of positive electrostatic potential.

**Figure 7:**
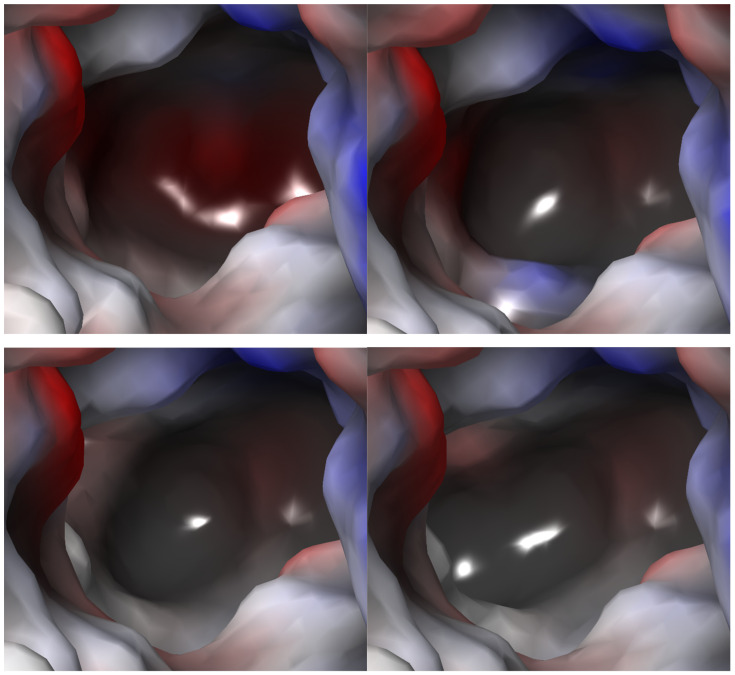
Electrostatic potential maps for the S1 binding site of taurine transporter mutants: Gly58Glu (**left, upper panel**), Leu306Gln (**right, upper panel**), Glu406Gly (**left, down panel**), and Glu406Cys (**right, down panel**). Red color—accumulation of negative electrostatic potential; blue color—accumulation of positive electrostatic potential.

Based on the constructed models and information from the literature, we compared the S1 binding sites of TauT, CT1 (model), and GAT1 (structure). It was observed that TauT, similarly to CT1, had a mostly hydrophobic S1 binding site (Figure 6). Like GAT1, they have electrostatic potential closer to zero comparing to other subfamily members [23]. Residues that lead to the difference between TauT and CT1 are Gly57, Leu134 and Glu406, and corresponding Phe68, Cys144 and Gly421, respectively. The presence of Glu406 increased the polarity of the TauT S1 site compared to CT1. Interestingly, Gly57, which exists in TauT, significantly reduced the polarity of the bottom of the S1 site, in comparison to the GAT2, GAT3 and BGT1, where glycine is replaced by a glutamate residue [23]. To better compare the change in electrostatic potential for selected residues in the SLC6A6 and GATs, we prepared models of the following mutants: Gly57Glu, Leu306Gln, Glu406Gly, and Glu406Cys (Figure 7). It was observed that Gly57Glu mutant caused significant changes in the electrostatic potential of the TauT binding site and modified its shape. This observation could be connected with reduced activity in taurine transport [28]. Leu306Gln mutant showed locally positive electrostatic potential, which may help in GABA binding. Changing Glu406 to glycine leads to decreased polarity and extended volume of the binding site (Figure 7). To further discuss the importance of selected residues for ligand binding, we performed docking studies. Initially, we conducted docking of taurine to the wild type of TauT. We also checked the binding mode of other taurine transporter ligands, like β-alanine, GABA, and hypotaurine. The importance of Glu406 was confirmed in docking to the mutant transporter models.

### 2.3. TauT Docking Studies

Docking studies allowed us to elucidate the binding mode of the main substrate, taurine, within the SLC6A6 transporter. Based on the models of TauT, GAT1 structure from the PDB database (PDB code: 7Y7W) as well as literature information about GABA transporters and CT1, we compared protein–substrate interactions, and identified residues crucial for ligand binding. Taurine was docked to ten homology models of the transporter selected based on DOPEScore, QMEAN and Verify3D scores. Additionally, we performed docking studies of other TauT substrates and inhibitors. Furthermore, we investigated the binding mode of the taurine within protein mutants to understand the impact of specific amino acid changes on ligand binding.

#### 2.3.1. TauT Ligands Binding Mode

**Figure 8:**
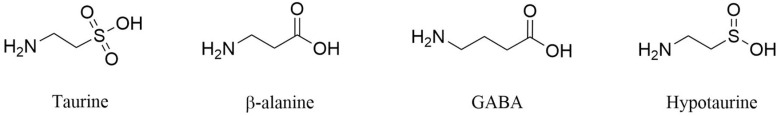
Structures of taurine transporter substrates [37].

Substrates of taurine transporter (Figure 8) possess a basic—amine—group and an acidic moiety—sulfonic (taurine), carboxy (β-alanine) or sulfinic—group (hypotaurine) connected by two carbon linkers. GABA, a “honorary” substrate of TauT, and main substrate of GAT1 possesses a three-carbon linker, which may interrupt its transport and interaction within the TauT. In comparison, a substrate of CT-1—creatine—has a methyl-guanidine moiety connected through one carbon linker with the carboxy group.

The general binding mode of taurine is similar to that of GABA with GAT1 and creatine with CT1 transporter: the acidic groups of ligands interact with Gly62 (TauT numeration), a hydroxy moiety of Tyr138 from the extracellular gate and sodium ions. The amine groups of substrates interact with phenylalanine from the extracellular gate (Phe300) and specific amino acids such as Glu406 in TauT, which may be important for substrate recognition.

**Figure 9:**
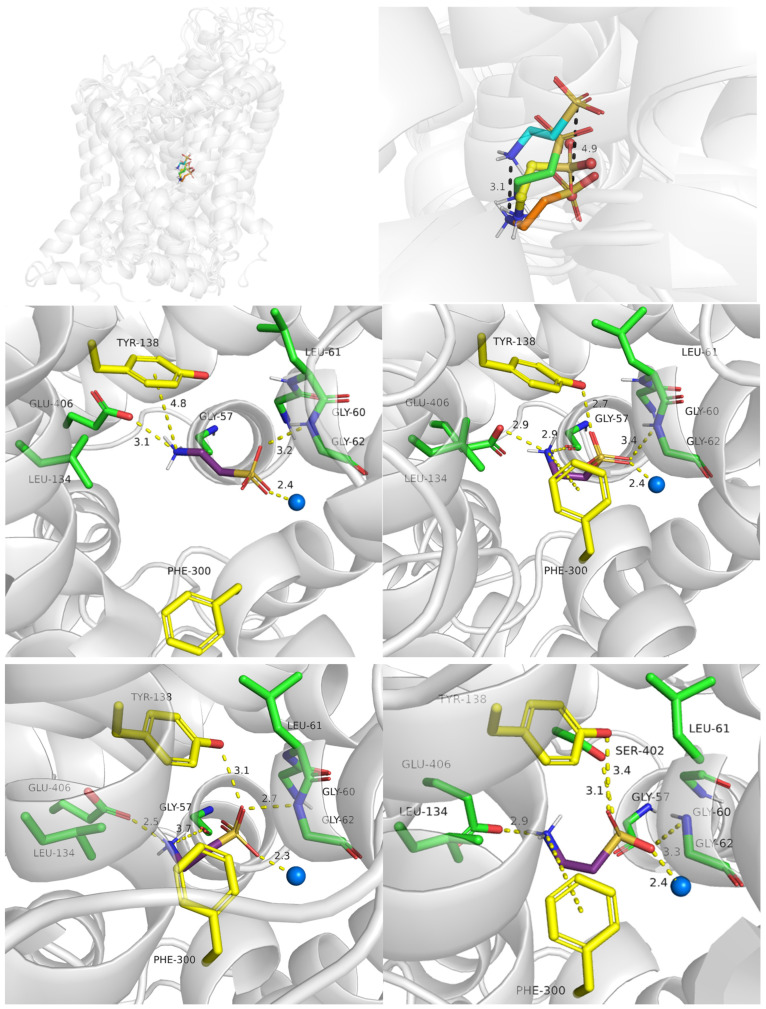
The overall structure of SLC6A6 with substrate—**upper panel**. Different colors of taurine represent its localization in: outward-open (blue), outward-occluded (green), inward-occluded (yellow), and inward-open (orange) states. Taurine binding mode within TauT: Outward-open state—**middle**, **left panel** (template: 4MMB, tool: SwissMODEL), outward-occluded state—**middle**, **right panel** (template: 2Q6H, tool: SwissMODEL), inward-occluded state—**down**, **left panel** (template: 7Y7W, tool: Modeller), and inward-open state—**down**, **right panel** (template: 7Y7Z, tool: Modeller). Residues color: yellow—extracellular gate, green—S1 site, blue sphere—sodium ion, and taurine—purple.

Herein, we present detailed information about the substrate binding modes obtained from in silico studies. Taurine reveals some shifts depending on the conformational state of the transporter. Comparing outward- and inward-open conformations, positions of acidic and basic groups are moved with the movement distance of 4.5 Å in the case of sulfur atom from the sulfonic group and 3.1 Å in the case of the nitrogen atom from the primary amine group (Figure 9, upper, right panel). Furthermore, taurine receives the following docking score values: −6.6, −7.2, −6.6 and −5.6 for outward-open, outward-occluded, inward-occluded, and inward-open states, respectively. Its binding modes at various steps of the transport are presented in Figure 9. First, in an outward-open state ionized amine group of taurine interacted with a specific TauT residue—Glu406—through a salt bridge (3.1 Å). It was also able to create cation–π interaction with Tyr138 (4.8 Å) from an extracellular gate. The sulfonate group of taurine created hydrogen bond with Gly62 (3.2 Å). This dissociated fragment was located near sodium ion and created with it ionic bond (Figure 9, middle, left panel). The hydrogen bond between Tyr138 and the acidic group of taurine was intended to create but due to distance above 4 Å it was very weak. Alternatively, it was possible that this interaction was mediated by a water molecule. Nevertheless, during domain movements accompanying transition to the next state it appeared. In the outward-occluded state, we observed a shorter distance between the amine group of the ligand and Glu406 (2.9 Å), which was connected with domain movements. Moreover, this group created cation–π interaction with the aromatic ring of Phe300 from the extracellular gate, which upon shift led to the reduction in the distance between the residue and the ligand. Taurine lost cation–π interaction with Tyr138 but revealed a hydrogen bond between Gly57 and a hydroxy group of tyrosine side chain (2.9 Å) (Figure 9, middle, right panel). In the inward-occluded state, similar interactions to those observed in the outward-occluded state were found (Figure 9, down, left panel). However, in the inward-open conformation, the distances between interacting groups were greater. Taurine interacted with Ser402 but lost the hydrogen bond with Gly57, due to the shift of TM1 domain (Figure 9, down, right panel).

**Figure 10:**
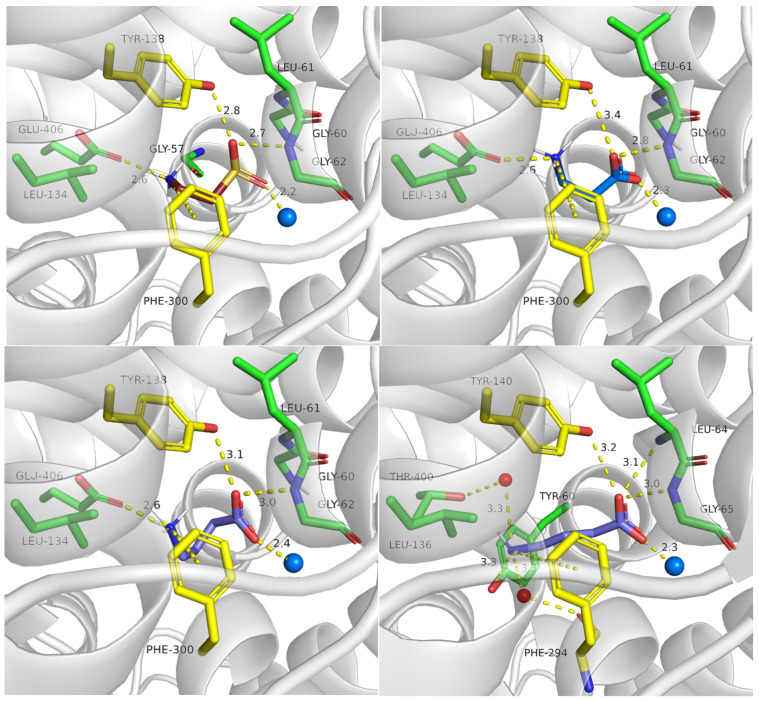
Binding mode of further substrates in an inward-occluded state of the taurine transporter (template: 7Y7W, tool: Modeller): hypotaurine (**left, upper panel**), β-alanine (**right, upper panel**), and GABA (**left, down panel**). For comparison, position of GABA in human GAT1 is shown (PDB code: 7Y7W; **right, down panel**). Residues color: yellow—extracellular gate, green—S1 site, blue sphere—sodium ion, red sphere—water molecules, hypotaurine—brown, β-alanine—marine, and GABA—slate.

Next, we analyzed binding of further substrates (hypotaurine, β-alanine and GABA) in different conformational states of the taurine transporter (Supplementary Materials: Figures S1–S3). Herein, we present the binding modes of these ligands in the inward-occluded state (Figure 10) to compare them with the available experimental complex of human GAT1 with bound GABA, obtained in the same conformation (Figure 10, right, down panel). The compounds obtained scoring function values as follows: −7.5 (hypotaurine), −7.0 (β-alanine), −5.7 (GABA). We observed that all ligands interacted within the S1 binding site similarly to the main substrate: their amine groups created ionic and cation–π interaction with Glu406 and Phe300, respectively. Sulfinic or carboxyl groups were near sodium ion and Gly62 (Figure 10). According to the fact that GABA has a longer alkyl linker than taurine, the ligand obtained not beneficial enough binding mode, which could explain lower affinity of GABA to the SLC6A6 than to the other GABA transporter subfamily member—GAT1 (Figure 10, both down panels).

#### 2.3.2. Binding of Taurine Transporter Inhibitors

**Figure 11:**
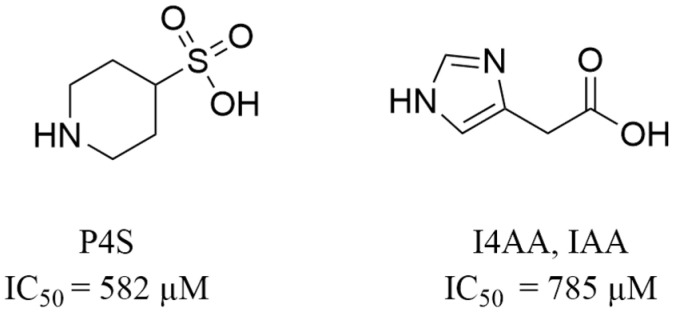
Structures of selected taurine transporter inhibitors. Their biological activity was determined by [^3^H]-taurine uptake using HEK293 cells producing TauT-GFP (green fluorescent protein) and expressed as IC_50_ values [37].

The taurine transporter represents a potential biological target for therapies, including anticancer treatment. Unfortunately, the pool of its inhibitors is limited. In our recent review, we discussed known ligands of taurine transporter [21]. According to literature findings, these compounds mimic the substrate—taurine. They typically feature amine groups like taurine, connected by aliphatic chains or rings with the acidic group [41,42,43]. Known inhibitors revealed various biological activity against SLC6A6, in the micromolar–millimolar concentration range. With further optimization, some of them could be tested against cancers, particularly colorectal cancer. Two inhibitors described in the literature, P4S (piperidine-4-sulfonic acid, IC_50_ = 582 µM) and I4AA (imidazole-4-acetic acid, IC_50_ = 785 µM) (Figure 11), have emerged as the most promising ligands alongside known taurine transporter substrates—β-alanine (IC_50_ = 44.5 µM), GABA (IC_50_ = 1014 µM) [41] and hypotaurine (13.3% [^3^H]-taurine uptake of control using 1 mM concentration screening) [44].

**Figure 12:**
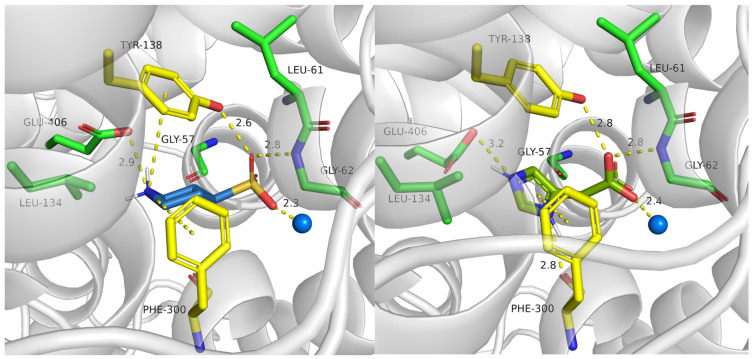
Binding mode of selected inhibitors in an inward-occluded state (template: 7Y7W, tool: Modeller) of the taurine transporter—P4S (**left panel**) and I4AA (**right panel**). Residues color: yellow—extracellular gate, green—S1 site, blue sphere—sodium ion, P4S—blue, and I4AA—green.

**Figure 13:**
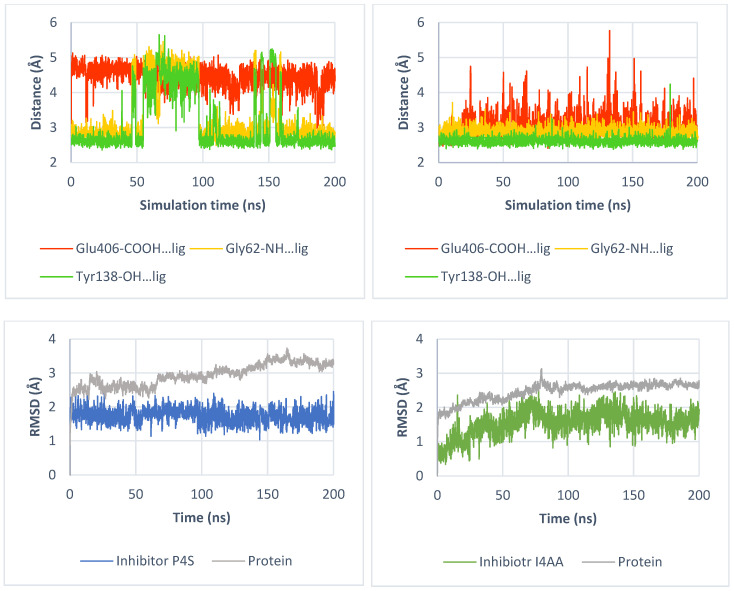
Distance changes between inhibitors and crucial amino acids: P4S (**left, upper panel**), I4AA (**right, upper panel**). Interactions color: orange; Glu406-COOH—ligand, yellow; Gly62-NH—ligand, green; Tyr138-OH—ligand. RMSD changes for protein and inhibitors: P4S (**left, down panel**) and I4AA (**right, down panel**) during molecular dynamics.

Here, we present the potential binding mode of P4S and I4AA in the inward-occluded state of SLC6A6, although compounds were docked to the four states. Generally, inhibitors of taurine transporter are small non-branched molecules, so they demonstrated favorable binding modes and interactions within the protein in the inward-occluded state (Figure 12). Both inhibitors, P4S and I4AA obtained beneficial scoring values of −8.5 and −8.8, respectively. The acidic groups of the inhibitors interacted with Tyr138, Gly62 and sodium ion, while Glu406 interacted with their basic groups. These residues appear to be crucial for substrate and inhibitor binding. The reliability of the presented binding modes of the inhibitors was further confirmed by 200 ns molecular dynamics simulations. Distance changes between functional groups of ligands and Glu406, Gly62 and Tyr138 were checked (Figure 13, upper left and right panel). It was noticed that the hydrogen bonds and ionic interaction of inhibitor I4AA were stable during the simulation. However, in the case of P4S, interactions between inhibitor and both Gly62 and Tyr138 were broken between 50 and 100 ns, although they were restored for the remaining simulation time. The average RMSD values for protein and P4S inhibitor were 2.46 Å and 1.7 Å, respectively (Figure 13, down, left panel). Similar RMSD values were noticed in the case of TauT and I4AA inhibitors: 2.93 Å and 1.5 Å, respectively (Figure 13, down, right panel). The inhibitory potency of these compounds could be related to the presence of the heterocyclic piperidine ring of P4S and the imidazole aromatic moiety of I4AA. These inhibitors possibly blocked TauT domain movements during transport due to their lower flexibility comparing to taurine. Literature analysis suggests that newly designed SLC6A6 inhibitors should contain an acidic moiety, such as the carboxyl, sulfonic, or phosphonic group (sulfate or phosphate are also acceptable) and the primary or secondary amine group connected by a small aliphatic or aromatic ring [21].

### 2.4. Role of Mutations in Taurine Transporter

#### 2.4.1. Glu406 Is Crucial for SLC6A6 Transporter Activity

To determine if Glu406 could be responsible for ligand selectivity, we compared the structure of taurine transporter with the structures of Glu406Gly and Glu406Cys mutants. We analyzed proteins in the outward-open conformation as the initial state with which ligands interact, and subsequently performed docking of substrates. Biological activity of Glu406Gly was not checked, whereas it is known that Glu406Cys mutant presented significant reduction in [^3^H]taurine uptake by 49% in comparison to the WT protein [28].

**Figure 14:**
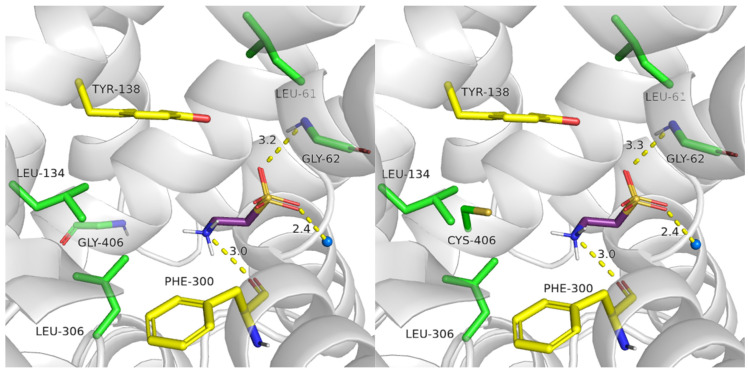
Binding mode of taurine within SLC6A6 mutants. Models were built based on the 4MMB template with SwissMODEL. Glu406Gly mutant (**left panel**); Glu406Cys (**right panel**). Residues color: yellow—extracellular gate, green—S1 site, blue sphere—sodium ion, and taurine—purple.

It was observed that taurine obtained a less beneficial docking score values in mutants (Glu406Gly: −5.46, Glu406Cys: −5.45) comparing to the scoring in the wild type of the transporter (−6.56). In the case of Glu406Gly mutant, taurine presented hydrogen bonds with Gly62, and the main chain of Phe300, but lost cation–π interaction with the aromatic ring of Tyr138. Additionally, taurine did not create ionic interaction by the amine group (Figure 14, left panel. Compared to Figure 9, left, middle panel). A similar observation was made for the Glu406Cys mutant (Figure 14, right panel). The lack of ionic or cation–π interactions with the amine group could impair the recognition of the substrate by TauT, resulting in decreased transporter activity [28].

#### 2.4.2. Importance of Gly57 and Leu306 for SLC6A6 Activity

**Figure 15:**
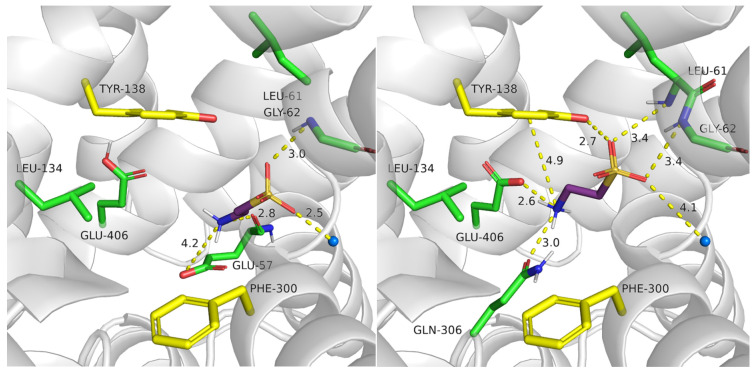
Binding mode of taurine within SLC6A6 mutants. Models were built based on the 4MMB template with SwissMODEL. Gly57Glu (**left panel**); Leu306Gln (**right panel**). Residues color: yellow—extracellular gate, green—S1 site, blue sphere—sodium ion, and taurine—purple.

Next, we evaluated Gly57Glu mutant. Biological studies showed that transporter with this mutation lost taurine uptake activity (reduced [^3^H]taurine uptake by of 99.8% in comparison to the WT). Docking to the Gly57Glu mutant, we observed an ionic interaction between the ligand and carboxy group of Glu57. However, the ligand was not able to create interaction with the aromatic ring of Tyr138 (Figure 15, left panel). In the Gly57Glu mutant, the carboxy group of Glu406 was predicted to be unionized. Possibly, the presence of an additional acidic group in the S1 site caused reduced dissociation of glutamic acid Glu406. Consequently, ligands did not interact with this residue via an ionic bond. This observation confirmed the importance of Gly57 and Glu406 for transporter functionality, as well as their essential role in ligand binding within the transporter.

Leu306 is also an important residue for TauT activity. In mutagenesis studies, Leu306Gln showed a significant reduction in [^3^H]taurine uptake by 97% in comparison to the WT protein. Our docking studies present a potential explanation for this effect [28]. Taurine in Leu306Gln mutant exhibited the same interactions, as these observed in the WT transporter in the outward-open state: acidic fragment interacted with Gly62 and sodium ion, the basic amine group formed a cation–π interaction with Tyr138 and Glu406. Moreover, hydrogen bonds with the hydroxy moiety of Tyr138, main chain of Leu61 as well as with Gln306 were observed. A change in the lipophilic residue Leu306 with polar glutamine led to the additional hydrogen bond and these interactions could stabilize the ligand-protein complex, and in consequence cause lower taurine transport in this mutant type. Moreover, the distance between the acidic group of taurine and sodium ion changed from 2.4 Å in the WT of transporter to 4.1 Å in the mutant. Due to the fact that the transport of taurine is sodium dependent, Leu306Gln mutation possibly has a negative impact on the protein activity.

## 3. Materials and Methods

### 3.1. Homology Modelling

The amino acid sequences of SLC6 family transporters were downloaded in FASTA format from the UniProt database [32]. These sequences were aligned using Clustal Omega [45] and compared with sequence alignments conducted by other researchers [23,24,25,26,27,28]. The resolution, sequence mutations, and conformational states of potential templates were considered during their selection for homology modelling. As a result, 14 experimental structures were selected. They included structures of: *Aquifex aeolicus* LeuT (*a*LeuT) in the outward-occluded state (PDB codes: 2A65, 2Q72, 2Q6H), and outward-open state (PDB codes: 3FAF, 4MMB, 4MM7); *Drosophila melanogaster* DAT (*d*DAT) in the outward-open state (PDB codes: 4XP4, 4XP9 6M2R), and outward-occluded state (PDB code: 4XPH); human SERT (*h*SERT) in the outward-open state (PDB code: 5I6X) and human GlyT1 (*h*GlyT1) in the inward-open state (PDB code: 6ZBV). These structures were obtained using X-ray diffraction, with resolution ranging from 1.65 to 3.40 Å. Additionally, *h*GAT1 structures in the inward-open state (PDB code: 7Y7Z), and inward-occluded state (PDB code: 7Y7W), obtained with cryogenic electron microscopy were used (Table S1). The sequences of the templates and SLC6A6 were aligned using BioEdit [46], omitting the N- and C- termini due to low sequence similarity. Homology modelling was conducted with the Modeller 9.15 program and SwissModel server [42]. Using MyModel class from Modeller, for each template, 200 models with high optimization levels were generated. Disulfide bridges were defined in the case of LeuT templates. In SwissModel, one model for each template was obtained. All models were evaluated with the DOPE, QMEAN scores and Verify 3D values. The best ten models were selected for further docking studies. The assessment of the selected models is presented in Table S2. Created models retained sodium and chloride ions from the templates during homology modelling if possible. Otherwise, to add the ions the models were superimposed onto ion bearing templates considering the residues from ion binding pockets—Phe58, Asn63, Ser301, Asn333, Gly56, Val59, Leu398, Asp401, Ser402, Tyr83, Gln297, Ser301, and Ser337—and then ions were copied to the models.

### 3.2. Docking Studies

The ligands were prepared with the Schrodinger Suite 2020-3 (Schrodinger Inc., New York, NY, USA), bond and chirality were checked. Compounds were ionized in physiological pH 7.4 ± 0.2 with Epic function from the LigPrep module. Selected models before docking were processed with the Protein Preparation Wizard module with default settings at pH 7.4 ± 0.2, including hydrogen bond assignment optimization and restrained minimization. For the best models, docking results were also checked without protein optimization. The grid for ligands docking was set as the inner box with edge length 15 × 15 × 15 (Å^3^) whereas the outer box size was 25 × 25 × 25 (Å^3^). Docking calculations were performed with standard precision, for each ligand 10 poses were written and sorted based on Docking Score values. The OPLS3e force field was used during the studies. In the case of taurine transporter inhibitors, the Induced Fit Docking module was employed. The centroid of the residues was determined based on the residues: Gly57, Gly60, Leu61, Gly62, Leu134, Ala303, Leu306, and Glu406. The results were analyzed using PyMOL and Maestro.

### 3.3. Molecular Dynamics Simulation

For molecular dynamics simulations, we employed Desmond from the Schrodinger Suite 2020-3 (Schrodinger Inc., New York, NY, USA). Initially, transporter models were positioned in the membrane using the OPM server [47]. Desmond molecular dynamics simulation protocol was as follows: the input files were prepared with the System Builder module, utilizing TIP3P water type and POPC membrane. The pre-aligned model with the OPM server was applied, and the size of the orthorhombic box was defined using the buffer method. The system was neutralized with chloride ions, and 0.15 M NaCl was added to obtain physiological conditions. The system was then minimized, and MD simulations were conducted under NPT condition. The simulation time was set to 200 ns with a recording interval of 100 ps, initiated from a random seed. For each inhibitor, three repeats of the simulation were performed.

## 4. Conclusions

Transporters from the SLC6 family play important physiological roles. In this study, we focused on the structure of the taurine transporter (SLC6A6), which is a potential biological target for anticancer therapy, due to its overexpression in colorectal, gastric, and cervical cancers [13,14,15]. Using a homology modelling approach, we obtained structural insight into the taurine transporter. Our structural analysis and docking studies were compared with literature data about other proteins from the GABA subfamily. We noticed that the overall structure of the taurine transporter closely resembles that of other SLC6 family members, featuring 12 transmembrane helices with N- and C-termini located intracellularly. The TM10 domain of SLC6A6 contained an additional residue Ser464, as observed in other GABA transporters. Based on the structural analysis, we proposed that this insertion forms a non-helical fragment. Homology modelling studies enabled us to obtain TauT models in different conformational states—outward-open, outward-occluded, inward-occluded and inward-open. We observed significant changes in the position of the TM1 domain, along with shifts in pairs of residues from the extracellular gate (Arg66–Asp469 and Tyr138–Phe300), as well as residues from the intracellular gate (Arg41–Asp416). Our docking studies of substrates and inhibitors provided insights into the crucial residues involved in ligand binding. We identified sodium ion and residues from the S1 site (Glu406, and Gly62), as well as residue from the extracellular gate (Tyr138), as pivotal for ligand binding. Glu406 took part in recognizing the amine group of taurine and formed ionic bond with it, while Gly62 and Tyr138 interacted via hydrogen bond with acidic fragment of the ligand. Docking studies with mutants of SLC6A6 (Glu406Gly, Glu406Cys) confirmed the importance of the Glu406 residue. The models generated in this study could serve as a basis for the design and development of novel SLC6A6 inhibitors, which could be further tested in cancer cell lines. By elucidating the structural features of TauT and its interactions with substrates, we provide valuable insights that could help in further work on taurine transporter inhibitors.

## Figures and Tables

**Table 1:** Classification of the SLC6 transporters.

Subfamilies	Human Gene Name	Transporter Name
GABA (γ-aminobutyric acid) transporter subfamily	SLC6A1 SLC6A6 SLC6A8 SLC6A10 SLC6A11 SLC6A12 SLC6A13	GAT1 TauT CT1 CT2 GAT3 BGT1 GAT2
Monoamine transporter subfamily	SLC6A2 SLC6A3 SLC6A4	NET DAT SERT
Glycine transporter subfamily	SLC6A5 SLC6A7 SLC6A9 SLC6A14	GlyT2 PROT GlyT1 ATB^0,+^
Neutral amino acid transporter subfamily	SLC6A15 SLC6A16 SLC6A17 SLC6A18 SLC6A19 SLC6A20	B^0^AT2 NTT5 NTT4 B^0^AT3 B^0^AT1 SIT1

SLC6A10 is known as a pseudogene [3].

**Table 2:** Sequence alignment for members of GABA subfamily and selected templates.

Sequences	*d*DAT		*a*LeuT		*h*SERT		*h*GlyT1		*h*GAT1	
Identity (I.), similarity (S.)	I. [%]	S. [%]	I. [%]	S. [%]	I. [%]	S. [%]	I. [%]	S. [%]	I. [%]	S. [%]
*h*GAT1	40.3	58.8	21.2	36.4	39.5	59.0	36.4	53.2	100.0	100.0
*h*BGT1	41.6	58.6	21.0	36.5	38.7	57.9	35.0	50.5	47.6	66.4
*h*GAT2	42.1	60.1	21.4	36.1	40.7	56.7	35.6	50.7	49.5	67.5
*h*GAT3	39.6	55.2	19.4	36.3	38.0	56.1	36.9	54.4	49.0	64.5
*hT*auT	41.2	58.2	21.5	34.1	38.1	53.4	35.8	53.6	46.5	63.1
*h*CT1	39.6	57.2	20.5	33.7	38.6	57.1	35.9	51.3	48.2	64.7

*d*DAT—*Drosophila melanogaster* dopamine transporter, *a*LeuT—*Aquifex aeolicus* leucine transporter, *h*SERT—human serotonin transporter, *h*GlyT—human glycine transporter and *h*GAT1–3—human γ-aminobutyric acid transporter 1–3, *h*BGT1—human betaine/GABA transporter, *h*TauT—human taurine transporter, *h*CT1—human creatine transporter 1.

**Table 3:** Residues from the extracellular gate and the S1 site of selected transporters from the SLC6 family (GABA transporter subfamily).

Protein	Extracellular Gate				S1 Site							
GAT1	Arg69	Tyr140	Phe294	Asp451	Tyr60	Gly63	Leu64	Gly65	Leu136	Gly297	Leu300	Thr400
BGT1	Arg61	Tyr133	Phe293	Asp452	Glu52	Gly55	Leu56	Gly57	Leu129	Ala296	Gln299	Cys399
GAT2	Arg57	Tyr129	Phe288	Asp447	Glu48	Gly51	Leu52	Gly53	Leu125	Ala291	Leu294	Cys394
GAT3	Arg75	Tyr147	Phe308	Asp467	Glu66	Gly69	Leu70	Gly71	Leu143	Ala311	Leu314	Cys414
TauT	Arg66	Tyr138	Phe300	Asp459	Gly57	Gly60	Leu61	Gly62	Leu134	Ala303	Leu306	Glu406
CT1	Arg77	Tyr148	Phe315	Asp474	Phe68	Gly71	Leu72	Gly73	Cys144	Ala318	Leu321	Gly421

## Data Availability

Data is contained within the article and Supplementary Materials.

## Supplementary Materials

The supporting information can be downloaded at: https://www.mdpi.com/article/10.3390/ijms25137339/s1. References [48,49,50,51,52,53] are cited in the Supplementary Materials.
