# Supplementary material for: Structural Studies of the Taurine Transporter: A Potential Biological Target from the GABA Transporter Subfamily in Cancer Therapy
Source: Int J Mol Sci. 2024 Jul 4;25(13):7339. doi: 10.3390/ijms25137339 (PMC11242302; doi:10.3390/ijms25137339)
Supplement: Supplementary file 1 [file ijms-25-07339-s001.zip › ijms-3057887-supplementary.pdf]

Structural studies of the taurine transporter: A potential biological target from the GABA transporter subfamily in cancer therapy

Dorota Stary [1,2] and Marek Bajda [1,*]

[1] Department of Physicochemical Drug Analysis, Faculty of Pharmacy, Jagiellonian University Medical College, Medyczna 9, 30-688 Cracow, Poland; dorota.stary@doctoral.uj.edu.pl

[2] Doctoral School of Medical and Health Sciences, Jagiellonian University Medical College, św. Łazarza 16, 31-530 Cracow, Poland

* Correspondence: marek.bajda@uj.edu.pl

Table S1. Protein from the PDB database selected as templates

Template	State	PDB code	Organism	Method	Res. (Å)	Date of release	Ref.
*a*LeuT	Outward-open	3FAF	*A. aeolicus*	X-ray diffraction	2.00	23.12.2008	[48]
		4MMB	*A. aeolicus VF5*	X-ray diffraction	2.25	16.10.2013	[49]
		4MM7	*A. aeolicus VF5*	X-ray diffraction	2.85	16.10.2013	
	Outward-occluded	2A65	*A. aeolicus VF5*	X-ray diffraction	1.65	02.08.2005	[26]
		2Q72	*A. aeolicus VF5*	X-ray diffraction	1.70	21.08.2007	
		2Q6H	*A. aeolicus VF5*	X-ray diffraction	1.85	21.08.2007	
*d*DAT	Outward-open	4XP4	*D. melan., M. musculus*	X-ray diffraction	2.80	06.05.2015	[50]
		4XP9	*D. melan., M. musculus*	X-ray diffraction	2.80	06.05.2015	
		6M2R	*D. melan., M. musculus*	X-ray diffraction	2.80	17.02.2021	[51]
	Outward-occluded	4XPH	*D. melan., M. musculus*	X-ray diffraction	2.90	06.05.2015	[50]
*h*SERT	Outward-open	5I6X	*H. sapiens., Mus mus.*	X-ray diffraction	3.14	13.04.2016	[38]
*h*GlyT1	Inward-open	6ZBV	*Homo sapiens*	X-ray diffraction	3.40	13.03.2021	[52]
*h*GAT1	Inward-occluded	7Y7W	*Homo sapiens*	Cryo-electron microscopy	2.40	26.04.2023	[53]
	Inward-open	7Y7Z	*Homo sapiens*	Cryo-electron microscopy	3.20	26.04.2023	[53]

A. aeolicus – Aquifex aeolicus, D. melan. – Drosophila melanogaster, M. musculus. – Mus musculus, H. sapiens – Homo sapiens. States were coloured: outward-open – blue, outward-occluded – green, inward-occluded – yellow, inward-open – orange.

Table S2. TauT model assessment with different tools

TauT transporter models							
Template	Tool	DopeScore	QMEAN	Verify-3D	Ramachandran plots – in the core	Ramachandran – not allowed	Residues in disallowed region
4MMB	SWISSMODEL	-79899.80	-5.43	70.62	89.7	0.0	-
2Q6H	SWISSMODEL	-81440.90	-6.18	75.33	90.02	0.4	Arg177, Ser188
4XP9	SWISSMODEL	-85320.80	-3.66	86.65	94.5	0.0	-
6M2R	Modeller	-81710.53	-3.34	91.96	94.3	0.0	Ser205, Arg423
4XPH	Modeller	-82012.69	-3.35	80.99	95.1	0.2	Ser205
4XPH	SWISSMODEL	-85195.60	-3.70	84.46	94.5	0.0	-
6ZBV	Modeller	-80891.83	-3.50	76.05	94.9	0.2	Ile184
6ZBV	SWISSMODEL	-83348.80	-4.28	74.13	92.3	0.2	Glu173
7Y7W	Modeller	-84508.70	-3.49	60.74	94.7	0.2	Asn533, Arg423
7Y7Z	Modeller	-82994.60	-4.11	63.52	93.2	0.2	Asp174, Ser187, Ser188, Thr189, Cys305, Asn533

States were coloured: outward-open – blue, outward-occluded – green, inward-occluded – yellow, inward-open – orange.

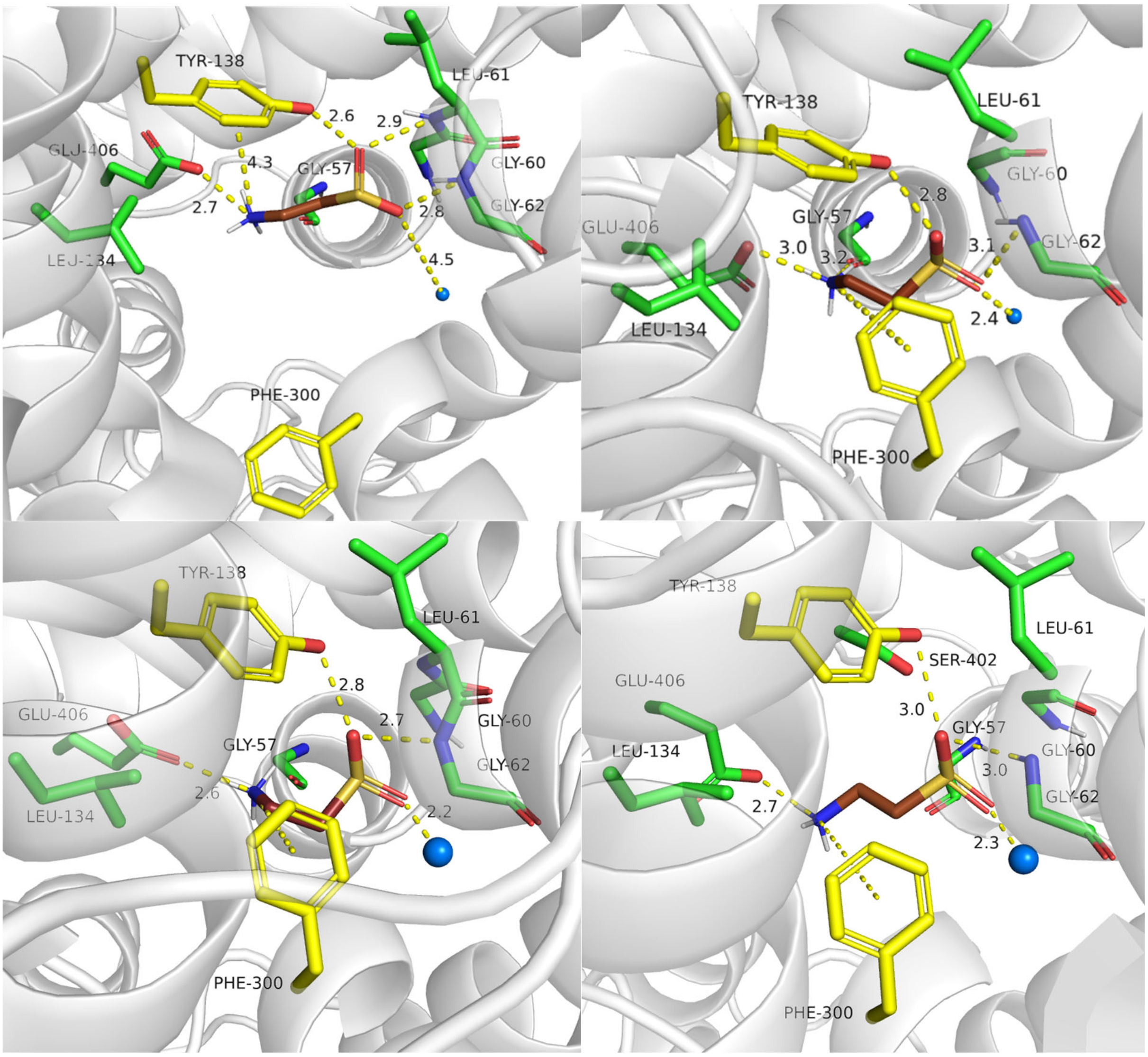

Figure S1. Hypotaurine bound to different conformational states of the taurine transporter: outward-open state - left, upper panel (template: 4MMB, tool: SwissMODEL), outward-occluded conformation – right, upper panel (template: 2Q6H, tool: SwissMODEL), inward-occluded state (template: 7Y7W, tool: Modeller), inward-open state (template: 7Y7Z, tool: Modeller). Hypotaurine obtained different scoring function values: -6.0 (outward-open state), -7.6 (outward-occluded state), -7.5 (inward-occluded state), -6.9 (inward-open state). Residues coloured: yellow – extracellular gate, green – S1 site, blue sphere – sodium ion, hypotaurine – brown

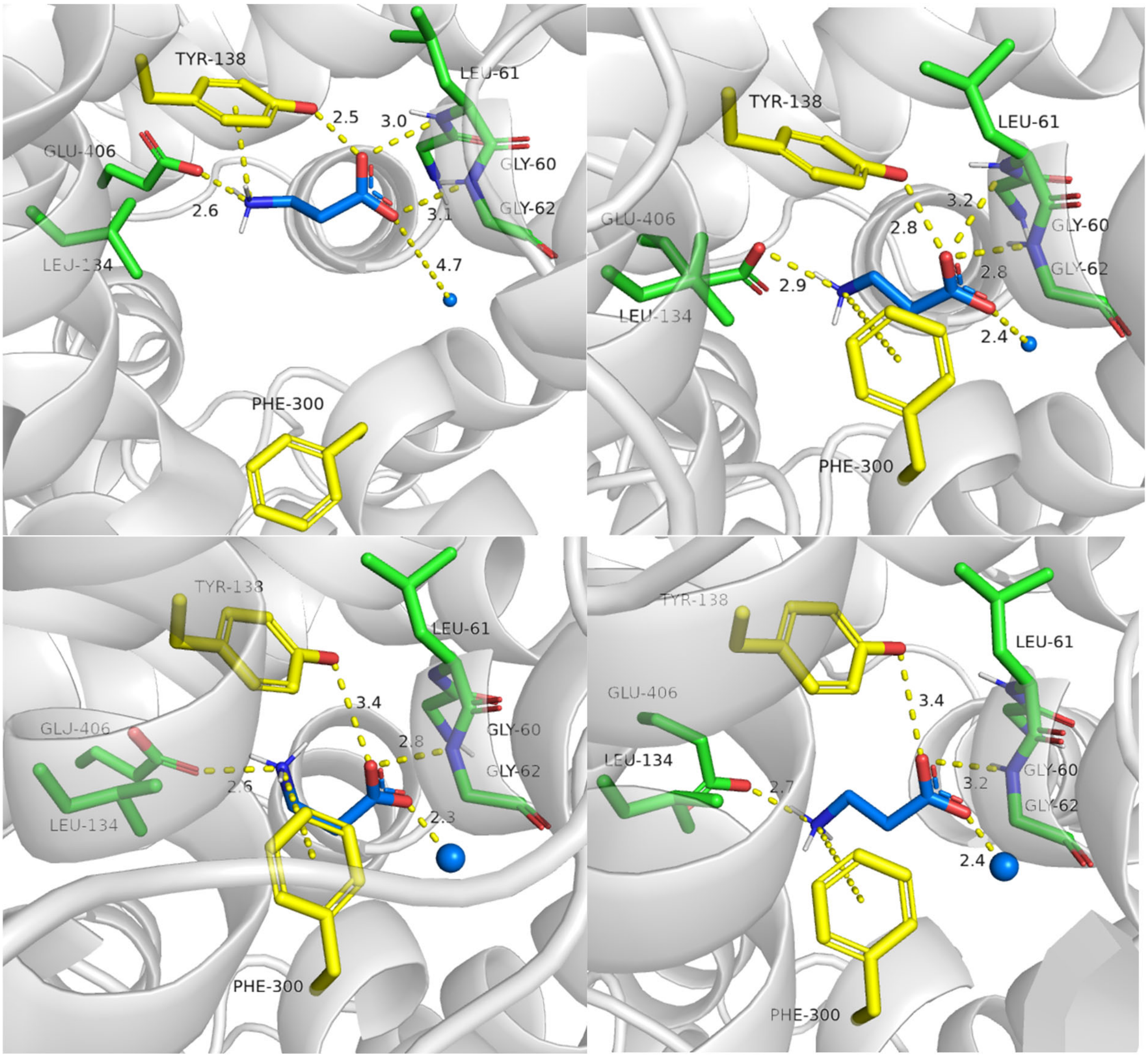

Figure S2. β-Alanine bound to different conformational states of the taurine transporter: outward-open state - left, upper panel (template: 4MMB, tool: SwissMODEL), outward-occluded conformation – right, upper panel (template: 2Q6H, tool: SwissMODEL), inward-occluded state (template: 7Y7W, tool: Modeller), inward-open state (template: 7Y7Z, tool: Modeller). β-Alanine obtained different scoring function values: -6.2 (outward-open state), -7.5 (outward-occluded state), -7.0 (inward-occluded state), -6.6 (inward-open state). Residues coloured: yellow – extracellular gate, green – S1 site, blue sphere – sodium ion, alanine – marine

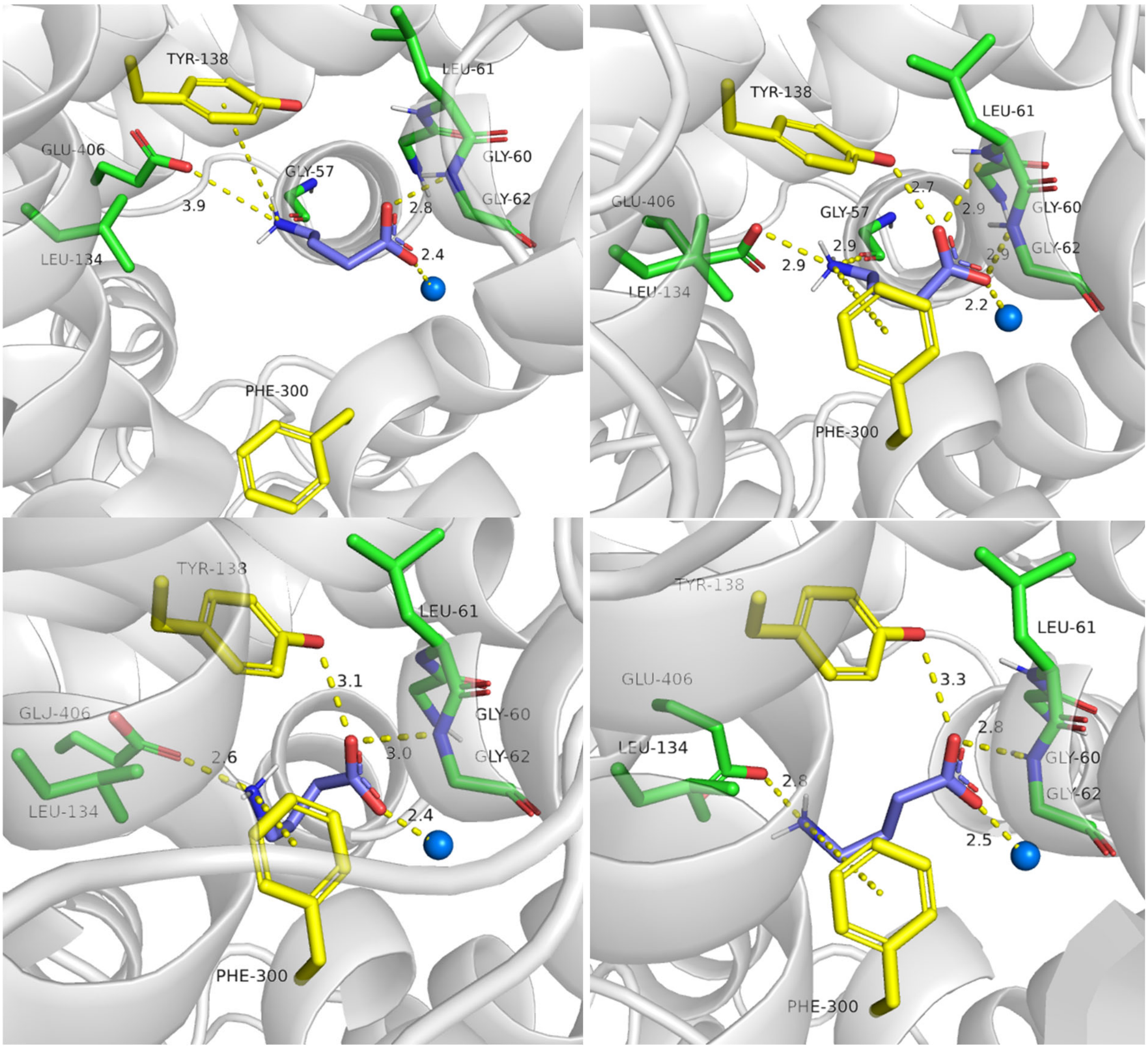

Figure S3. GABA bound to different conformational states of the taurine transporter: outward-open state - left, upper panel (template: 4MMB, tool: SwissMODEL), outward-occluded conformation – right, upper panel (template: 2Q6H, tool: SwissMODEL), inward occluded state (template: 7Y7W, tool: Modeller), inward-open state (template: 7Y7Z, tool: Modeller). GABA obtained different scoring function values: -5.5 (outward-open state), -7.7 (outward-occluded state), -5.7 (inward-occluded state), -5.2 (inward-open state). Residues coloured: yellow – extracellular gate, green – S1 site, blue sphere – sodium ion, GABA-slate
